# NOD2 inhibits tumorigenesis and increases chemosensitivity of hepatocellular carcinoma by targeting AMPK pathway

**DOI:** 10.1038/s41419-020-2368-5

**Published:** 2020-03-06

**Authors:** Xiaomin Ma, Yumin Qiu, Yanlin Sun, Lihui Zhu, Yunxue Zhao, Tao Li, Yueke Lin, Dapeng Ma, Zhenzhi Qin, Caiyu Sun, Lihui Han

**Affiliations:** 1 Shandong Provincial Key Laboratory of Infection & Immunology, Department of Immunology, Shandong University School of Basic Medical Sciences, Jinan, 250012 China; 2 Department of Pathology, Shandong University School of Basic Medical Sciences, Jinan, 250012 China; 3 Department of Pharmacology, Shandong University School of Basic Medical Sciences, Jinan, 250012 China; 4 Department of Gastroenterology, Provincial Hospital Affiliated with Shandong University, Jinan, 250021 China

**Keywords:** Liver cancer, Macroautophagy, Tumour-suppressor proteins

## Abstract

Nucleotide binding oligomerization domain 2 (NOD2) is a recognized innate immune sensor which can initiate potent immune response against pathogens. Many innate immune sensors have been reported to be of great importance in carcinogenesis. However, the role of NOD2 in cancer is not well understood. Here we investigated the role of NOD2 in the development of hepatocellular carcinoma (HCC). We demonstrated that NOD2 deficiency promoted hepatocarcinogenesis in *N*-nitrosodiethylamine (DEN)/carbon tetrachloride (CCl_4_) induced HCC mice model and xenograft tumor model. In vitro investigation showed that NOD2 acted as a tumor suppressor and inhibited proliferation, colony formation and invasion of HCC cells. Clinical investigation showed that NOD2 expression was completely lost or significantly downregulated in clinical HCC tissues, and loss of NOD2 expression was significantly correlated with advanced disease stages. Further investigation showed that NOD2 exerted its anti-tumor effect through activating adenosine 5′-monophosphate (AMP) -activated protein kinase (AMPK) signaling pathway, and NOD2 significantly enhanced the sensitivity of HCC cells to sorafenib, lenvatinib and 5-FU treatment through activating AMPK pathway induced apoptosis. Moreover, we demonstrated that NOD2 activated AMPK pathway by directly binding with AMPKα-LKB1 complex, which led to autophagy-mediated apoptosis of HCC cells. Altogether, this study showed that NOD2 acted as a tumor suppressor as well as a chemotherapeutic regulator in HCC cells by directly activating AMPK pathway, which indicated a potential therapeutic strategy for HCC treatment by upregulating NOD2-AMPK signaling axis.

## Introduction

Hepatocellular carcinoma (HCC) is one of the most common malignancies worldwide with increased morbidity and mortality in recent years. The therapeutic strategy for HCC is quite limited due to the common presence of resistance to chemotherapy^1,2^. Because of the critical connection between adenosine 5′-monophosphate (AMP)-activated protein kinase (AMPK) pathway and multiple cancers related signaling including mammalian target of rapamycin complex 1 (mTORC1) pathway, AMPK pathway is recognized to play a pivotal role in cancer^3^. Deficiency of AMPK pathway is reported to contribute to the progression of cancer and resistance to chemotherapeutic drugs in multiple types of cancers^4–6^. However, the cause of dysregulation of AMPK pathway in cancer and its molecular mechanism remains to be clarified. In this study, we identified nucleotide binding oligomerization domain 2 (NOD2), an innate immune sensor, as an efficient direct regulator of AMPK pathway; and loss of its expression in cancer cells promoted HCC progression and resistance to chemotherapy.

NOD2 is a member of NOD-like receptor family, and it is recognized as a sensor of the bacterial peptidoglycan (PGN)-conserved motifs in cytosol to stimulate subsequent innate immune responses^7^. However, many recent studies indicated that innate immune sensors, including nucleotide-binding domain, leucine-rich family (NLR), pyrin-containing 3 (NLRP3) and absent in melanoma 2 (AIM2) were also involved in carcinogenesis^8–10^. Dysregulation of NOD2 was reported to be involved in the pathogenesis of Crohn’s disease (CD) and colitis related colon cancer^7^; and NOD2 gene polymorphisms have been associated with increased risk of lymphoma, colorectal, gastric, breast, ovarian, lung, and laryngeal cancers^11^. Furthermore, it is reported that NOD2 agonists can activate the cytotoxic potential of immune cells residing in the tumor microenvironment (TME) and, consequently facilitate their engagement with cancer cells^12^. However, the exact role of NOD2 in HCC is still far from being clarified.

In this study, we showed that innate immune sensor NOD2 acted as a tumor suppressor in HCC progression and inhibited hepatic tumorigenesis in vivo and in vitro. We also showed that NOD2 significantly reversed the resistance of HCC cells to chemotherapeutic drugs. Further investigation demonstrated that NOD2 could activate AMPK pathway by directly binding with serine/threonine-protein kinase STK11 (LKB1)–AMPK complex and delicately regulating the LKB1/AMPK pathway, which further induced subsequent autophagy-mediated apoptosis of HCC cells. Thus, our investigation provided a novel clue for clarifying the molecular mechanism involved in disease progression and response to chemotherapeutic drugs in HCC, which indicated an optional therapeutic strategy for HCC treatment by modulating NOD2/AMPK signaling axis.

## Results

### NOD2 deficiency promoted hepatocarcinogenesis in NOD2 null mice

**Fig. 1:**
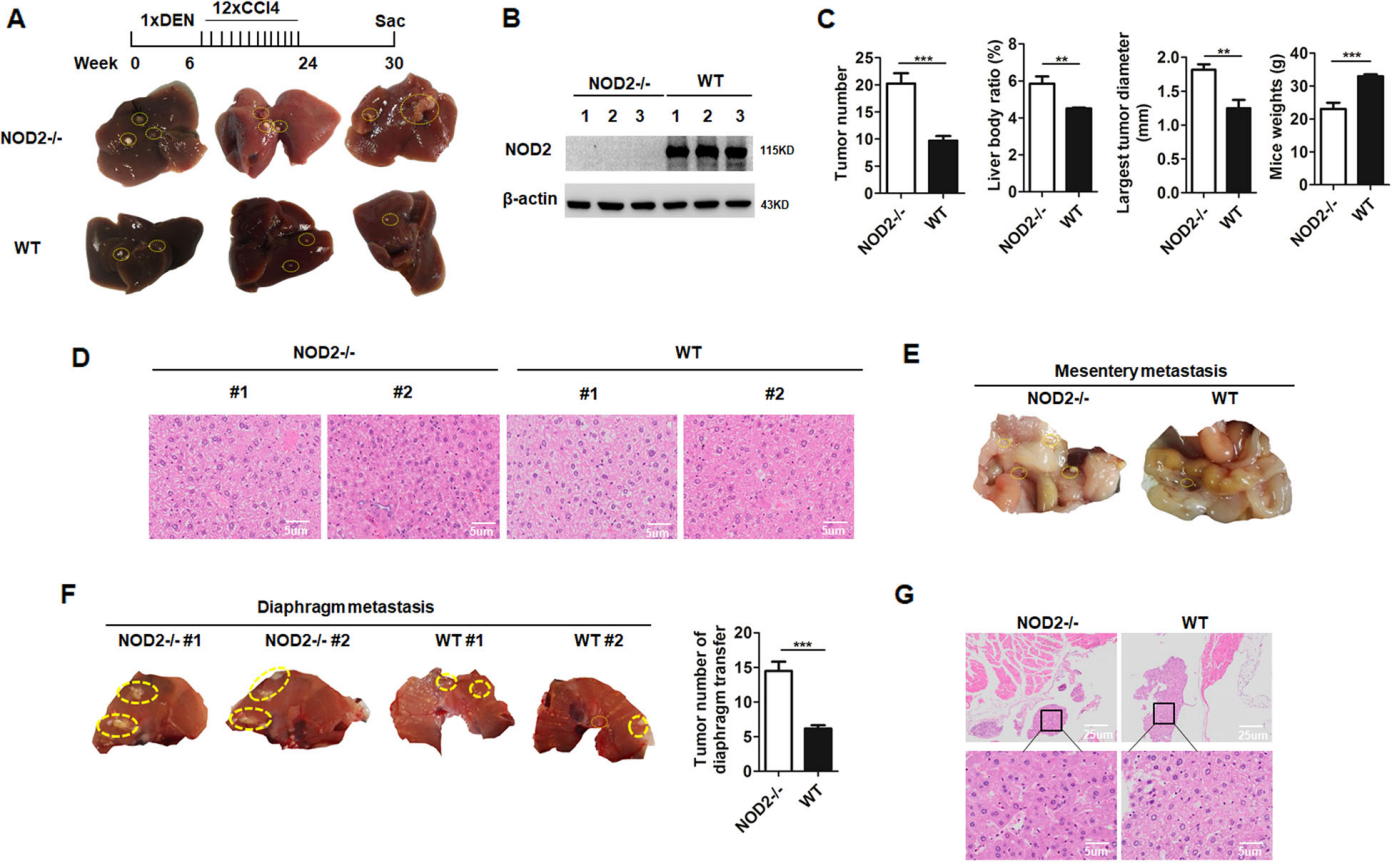
NOD2 deficiency promoted hepatocarcinogenesis in vivo. NOD2-/- mice (*n* = 7) and WT mice (*n* = 8) were injected with DEN (100 mg/kg, i.p.) at the age of 6 weeks followed by 12 injections of CCl_4_ (0.5 ml/kg, i.p.). The mice were sacrificed at 24 weeks after DEN injection. **a** Formed tumors in livers separated from the NOD2-/- mice and WT mice were presented. **b** NOD2 expression of the isolated livers from NOD2-/- and WT mice was detected by western blot assay. **c** Tumor numbers, liver body ratios, the largest tumor diameters and body weights of the NOD2-/- and WT mice were analyzed and compared. **d** HE staining of the liver tissues from NOD2-/- and WT mice was presented. **e, f** Tumor metastasis loci in mesentery (**e**) and diaphragm (**f**) from NOD2-/- and WT mice were quantitatively analyzed and compared. **g** HE staining of the diaphragmatic tumor metastases from NOD2-/- and WT mice was presented. **P < 0.01, ***P < 0.001 for statistical analysis of the indicated groups.

To verify whether NOD2 played a role in HCC progression, we constructed HCC animal model by injecting *N*-nitrosodiethylamine (DEN) and carbon tetrachloride (CCl_4_) to NOD2-/- and WT mice multiple times as indicated in Fig. 1a according to the reference^13^. The mice were sacrificed 24 weeks after the induction, and the tumor formation status of WT and NOD2-/- mice were analyzed and compared (Fig. 1a). NOD2 deficiency in NOD2-/- mice was confirmed by western blot (Fig. 1b). Compared with the WT mice, the tumor numbers, liver body ratios, and tumor diameters in the NOD2-/- mice were significantly increased (Fig. 1a, c), whereas the body weights of the NOD2-/- mice were significantly decreased (Fig. 1c). HE staining showed that tumors in the NOD2-/- mice were less differentiated compared with the WT mice (Fig. 1d). Furthermore, the NOD2-/- mice presented more metastasis loci in the mesentery (Fig. 1e) and the diaphragm (Fig. 1f) than those in the WT mice, and these metastatic loci were further confirmed by pathological analysis by two professional pathologists (Fig. 1g). These data collectively indicated that NOD2 deficiency facilitated hepatocarcinogenesis and exacerbated HCC development.

### NOD2 suppressed malignancy of HCC cells in vivo and in vitro

**Fig. 2:**
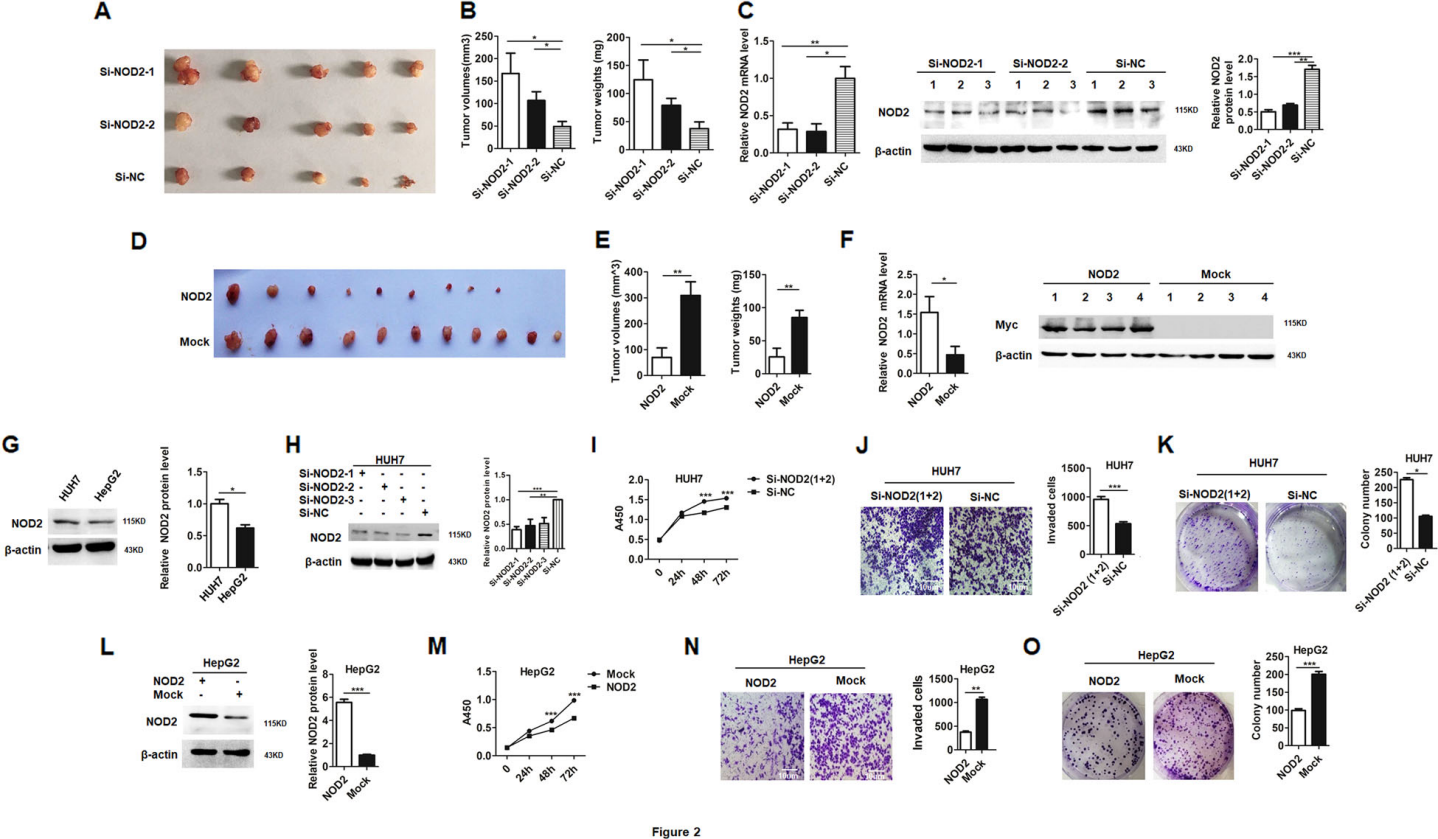
NOD2 suppressed xenograft tumor growth and the malignant behaviors of HCC cells. **a** 1 × 10^7^ HUH7 cells were subcutaneously injected to the right flanks of the nude mice (*n* = 15). When visible tumor appeared, the mice were randomly divided into three groups, and the investigator was blinded to the group allocation. Two groups of tumors (*n* = 5 for each group) were injected with two independent siRNAs against NOD2 (Si-NOD2), and the other group (*n* = 5) was injected with nonsense sequence (Si-NC) as a control. Images of the isolated tumors from the three experimental groups were presented. **b** Tumor volumes (left panel) and tumor weights (right panel) of these three groups of tumors were measured and statistically analyzed. **c** Relative NOD2 mRNA expression in these groups was detected by qRT-PCR (left panel). The relative NOD2 protein expression was detected by western blot (middle panel), and band intensities were statistically analyzed (right panel). **d** 1 × 10^7^ HUH7 cells were subcutaneously injected to both flanks of the nude mice (*n* = 11). When visible tumor appeared, the tumors in the left flanks were transfected with NOD2 plasmid while tumors in the right flanks were transfected with mock plasmid. Images of the tumors separated from both flanks of the nude mice were presented. **e** Tumor volumes and tumor weights of NOD2 and mock plasmid transfected groups were statistically analyzed and compared. **f** qRT-PCR (left panel) and western blot (right panel) were performed to detect the relative NOD2 expression level in NOD2 plasmid and mock plasmid transfected group. **g** The basal expression levels of NOD2 in two HCC cell lines were detected by western blot assay. **h** Three interference RNAs against NOD2 were synthesized and tested in HUH7 cells for their blocking efficiency. **i** HUH7 cells were transfected with the mixture of Si-NOD2-1 and Si-NOD2-2, and the proliferation of HCC cells was detected at 0 h, 24 h, 48 h and 72 h. **j**, **k** HUH7 cells were transfected with the mixture of Si-NOD2-1 and Si-NOD2-2, and the invasion (**j**) and colony formation capabilities (**k**) of these transfected cells were detected and compared. **l–o** HepG2 cells were transfected with NOD2 plasmid or mock plasmid, western blot was performed to verify the successful overexpression of NOD2 (**l**). The proliferation of HCC cells was detected at 0 h, 24 h, 48 h and 72 h after the transfection (**m**). Invasion (**n**) and colony formation capabilities (**o**) of these transfected cells were detected and statistically analyzed. *P < 0.05, **P < 0.01, ***P < 0.001 for statistical analysis of the indicated groups. Presented figures are representative data from three independent experiments.

To further verify the role of NOD2 in HCC, xenograft tumor models were constructed as described before^14^. After visible tumors appeared, the formed tumors were transfected with specific small interference RNA (SiRNA) against NOD2 (Si-NOD2) to construct the loss-of- function model in vivo according to the protocol described before^15^. The mice were sacrificed on day 38 after the first transfection, and the formed tumors were statistically analyzed. Both the volumes and weights of the formed tumors were dramatically increased in the Si-NOD2-transfected groups compared with the Si-NC transfected group (Fig. 2a, b). Real-time polymerase chain reaction (PCR) and western blot assay demonstrated successful knockdown of NOD2 in the Si-NOD2-transfected groups (Fig. 2c), which verified that loss of NOD2 expression exacerbated hepatocarcinogenesis. In another parallel set of experiment, the formed tumors were transfected with NOD2 expression plasmid to construct the gain-of-function model in nude mice. The mice were sacrificed and the tumors were isolated on day 21 after the first injection of the plasmid. Among these formed tumors, two tumors in the NOD2-transfected group completely disappeared. Further assay showed that tumors transfected with NOD2 plasmid had significantly smaller tumor volumes and lower tumor weights than those tumors transfected with the mock plasmid (Fig. 2d, e). Real-time PCR and western blot assay verified successful overexpression of NOD2 in the NOD2 plasmid transfected group (Fig. 2f). Thus these in vivo data indicated that NOD2 inhibited the xenograft tumor growth in HCC mice models.

To verify the anti-tumor effect of NOD2 on HCC, the function of NOD2 in HCC cellular models was further analyzed. We constructed gain-of-function cellular model by transfection of NOD2 plasmid to HepG2 cells which had a lower level of NOD2 expression, and we further constructed loss-of-function model by transfection of Si-NOD2 to HUH7 cells which had a higher level of NOD2 expression (Fig. 2g). In the loss-of-function model, efficient knockdown of NOD2 by three Si-NOD2 sequences was verified by western blot (Fig. 2h), therefore the following NOD2 knockdown experiment was done with the mixture of two Si-NOD2 sequences with higher inhibitory efficiency. Our data showed that after knockdown of NOD2, the proliferation, invasion and colony formation capabilities of HCC cells were significantly increased (Fig. 2i–k), while overexpression of NOD2 in HCC cells inhibited these malignant behaviors (Fig. 2l–o). As muramyl dipeptide (MDP) is a recognized ligand as well as an agonist of NOD2 in innate immune response^16^, we were interested in defining whether MDP had the same anti-tumor effect as NOD2. Our data showed that in agreement with the effect of exogenous NOD2 overexpression, MDP treatment could also inhibit the malignant behaviors of HCC cells (Figure S1A–C). Altogether, these in vitro data further supported our data from animal models that NOD2 acted as a tumor suppressor in HCC progression.

### Absent expression of NOD2 contributed to clinical HCC progression

**Fig. 3:**
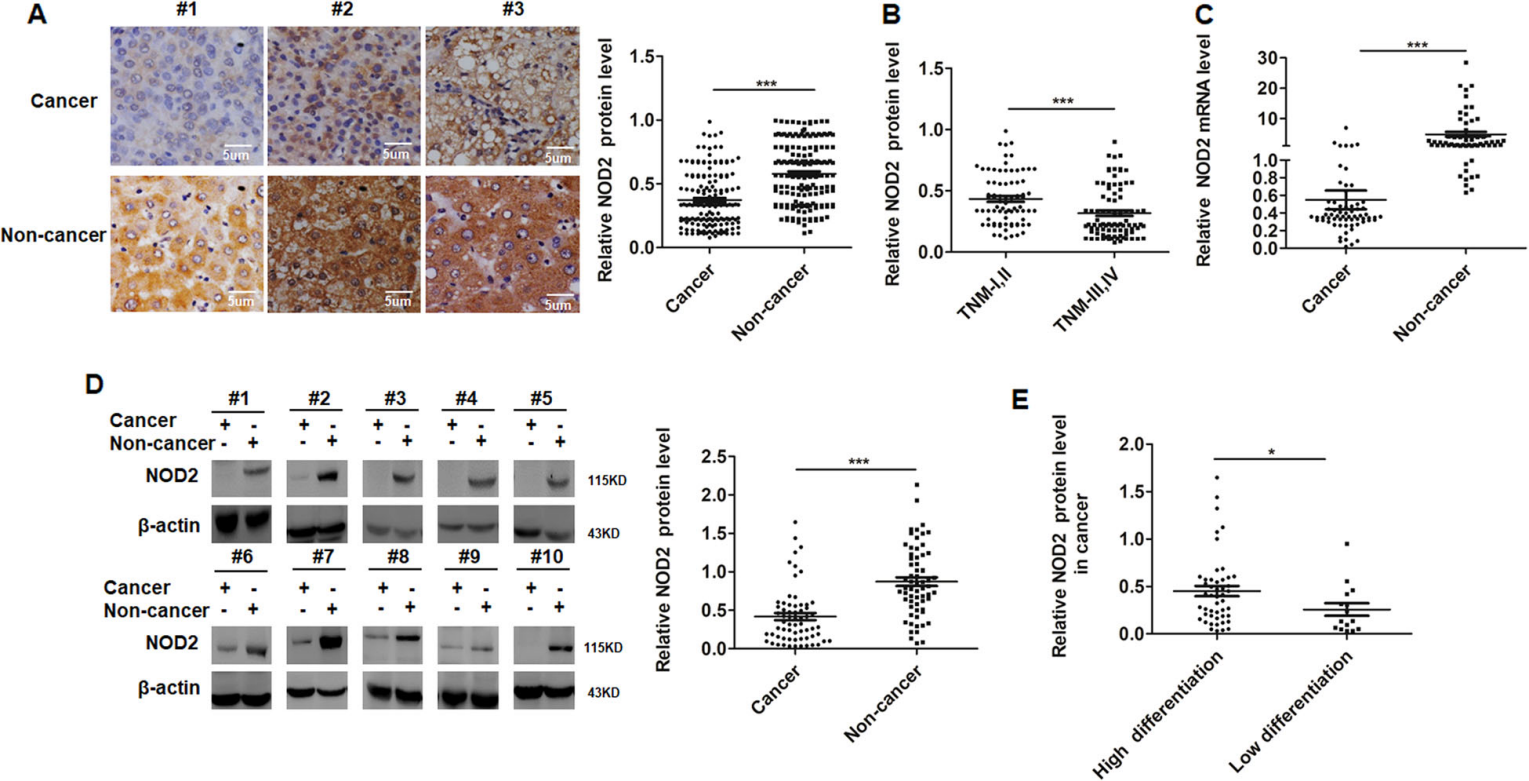
Absent expression of NOD2 contributed to clinical hepatocellular carcinoma progression. **a** 165 pairs of HCC tissues and their corresponding non-cancerous liver tissues were used for IHC assay to detect the location and relative expression of NOD2. Presented images were representative figures of the investigated HCC cases (left panel). NOD2 expression by IHC assay in cancerous and non-cancerous liver tissues was further quantitatively analyzed by IPP6 software (right panel). **b** NOD2 expression in different TNM stages by IHC assay was statistically analyzed. **c** Expression levels of NOD2 mRNA in 64 HCC tissues and matched non-cancerous liver tissues were detected and statistically analyzed. **d** Western blot was performed to detect the relative protein levels of NOD2 in paired HCC samples and representative bands were presented (left panel). The relative band densities from all of the detected patients were analyzed by Image J software and normalized by β-actin (right panel). **e** The relative NOD2 protein expression in well-differentiated tumors and poor-differentiated tumors were statistically analyzed. *P < 0.05, **P < 0.01 and ***P < 0.001 for statistical analysis of the indicated groups.

To demonstrate the role of NOD2 in clinical HCC progression, NOD2 expression was detected by immunohistochemistry (IHC) in one cohort including 165 pairs of HCC tissues and corresponding distal non-cancerous liver tissues; followed by western blot and real-time PCR assay of the NOD2 expression in another cohort of 64 patients with matched pairs of HCC tissues and distal non-cancerous liver tissues. The clinicopathologic features of all these recruited HCC patients were presented in Supplementary Table 1. IHC assay indicated that NOD2 expression was significantly decreased in HCC tissues compared with matched non-cancerous liver tissues (Fig. 3a, Supplementary Table 2), average optical density analysis showed the same outcome (Fig. 3a). Further analysis of the IHC staining showed that NOD2 expression was dramatically decreased in HCC patients with advanced tumor node metastasis (TNM) stages (Fig. 3b). In consistence with the IHC data, quantitative real time PCR (qRT-PCR) and western blot assay also showed that NOD2 expression was either completely lost or significantly decreased in HCC tissues (Fig. 3c, d). Further statistical analysis showed that HCC patients with poorer differentiation status were prone to have significantly lower level of NOD2 expression compared with the well differentiated HCC patients (Fig. 3e). In consideration of the tumor suppressor role of NOD2 in animal and cellular models, all these clinical data indicated that loss of NOD2 expression in HCC patients contributed to HCC progression.

### NOD2 inhibited HCC progression through activating AMPK signaling pathway

**Fig. 4:**
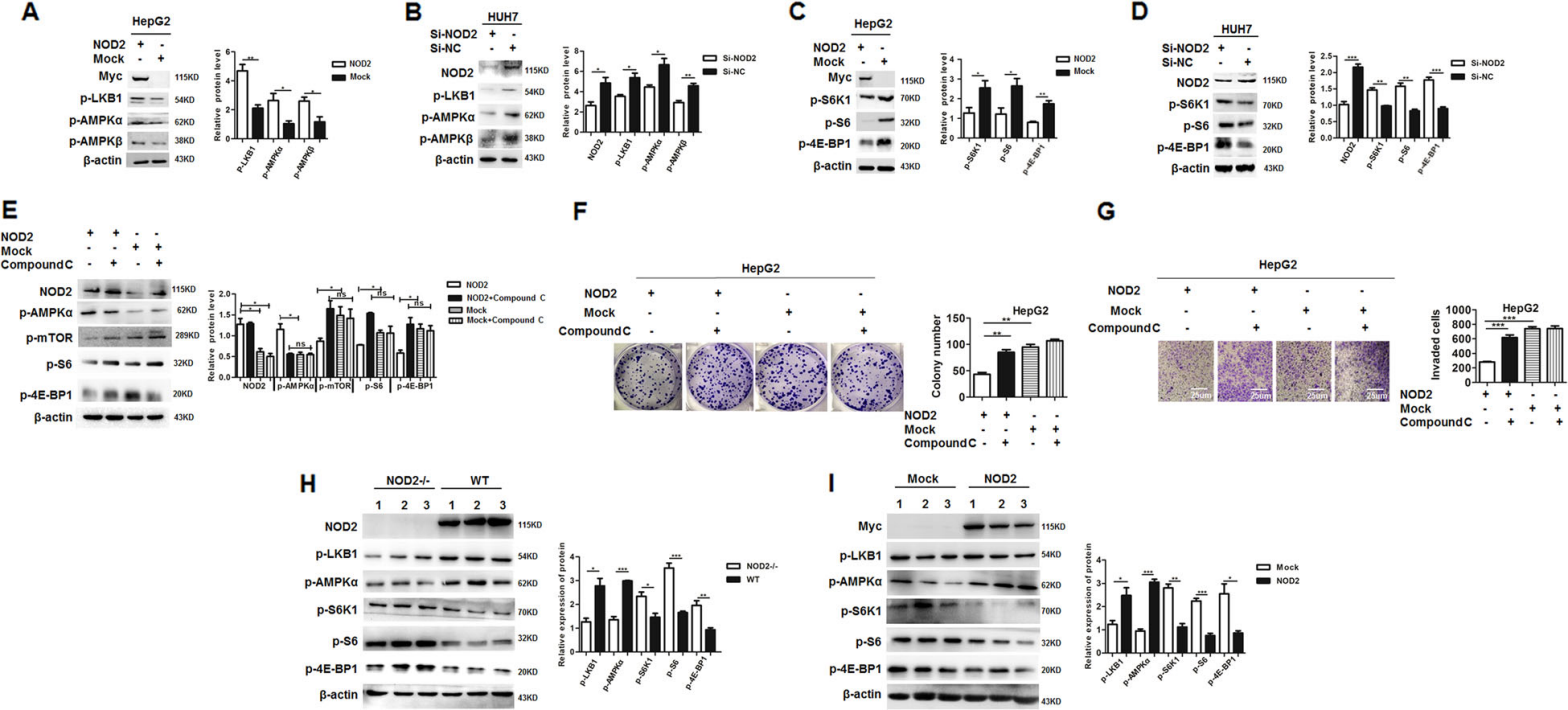
NOD2 inhibited HCC progression through activating AMPK signaling pathway. **a** HepG2 cells were transfected with NOD2 plasmid or mock control plasmid, and further cultured for 24 h. Activation status of AMPK pathway, including p-LKB1, p-AMPKα and p-AMPKβ were detected by western blot. **b** HUH7 cells were transfected with Si-NOD2 or Si-NC, and the activation status of AMPK pathway was detected by western blot. **c** HepG2 cells were transfected with NOD2 plasmid or mock control plasmid, and the activation status of mTORC1 pathway, including p-S6K1, p-S6 and p-4E-BP1 were detected by western blot. **d** HUH7 cells were transfected with the mixture of two SiRNAs against NOD2 (Si-NOD2), and the cells transfected with random sequences (Si-NC) acted as a mock control. Activation status of mTORC1 pathway was detected by western blot. **e**–**g** HepG2 cells were transfected with NOD2 plasmid, and further cultured for 6 h before compound C (10 μM) was added to inhibit the activation of AMPK pathway. Western blot assay was performed to detect the expression of NOD2, p-mTOR, p-AMPKα, p-S6K1, p-S6 and p-4E-BP1 after the transfection (**e**). Colony formation (**f**) and invasion (**g**) of the NOD2-transfected HCC cells with compound C treatment were analyzed. **h** NOD2-/- and WT mice were injected with DEN and CCl_4_ to induce hepatocellular carcinoma, and western blot assay was performed to detect the AMPK-mTORC1 pathway activation in the liver tissues from the NOD2-/- and WT mice. **i** HCC cells were injected to the nude mice to construct the xenograft tumor models as described before. When visible tumor appeared, the mice were divided into NOD2-transfected group and the mock control group. Relative expression of the related signaling transduction pathway proteins, including AMPK pathway related proteins and mTORC1 pathway related proteins were detected by western blot. The densities of all bands were analyzed by Image J software and statistically analyzed by GraphPad software. *P < 0.05, **P < 0.01, ***P < 0.001 for statistical analysis of the indicated groups.

To clarify the signaling transduction pathway of NOD2 in HCC cells, several possible involved pathways were tested and all of these data indicated the involvement of AMPK pathway in the effect of NOD2 on HCC cells. After overexpression of NOD2, the activation of AMPK pathway related proteins including p-LKB1, p-AMPKα and p-AMPKβ was significant upregulated (Fig. 4a), which indicated dramatically activation of AMPK pathway. In consistence with the gain-of-function data, knockdown of NOD2 expression significantly inhibited the phosphorylation and activation of AMPK pathway (Fig. 4b).

It is recognized that mTORC1 pathway is at the downstream of AMPK pathway and is suppressed by the AMPK pathway^17^, thus the activation status of mTORC1 pathway was further detected in HCC cells. As expected, overexpression of NOD2 in HCC cells inhibited mTORC1 pathway (Fig. 4c), while knockdown of NOD2 promoted the activation of mTORC1 signaling (Fig. 4d). When AMPK pathway was blocked by its specific inhibitor Compound C, the inhibition of mTORC1 pathway by NOD2 was significantly reversed (Fig. 4e). Thus it verified that inhibition of mTORC1 pathway by NOD2 was at the downstream of AMPK pathway. Furthermore, the inhibitory effect of NOD2 on the malignant behaviors of HCC cells was also significantly rescued by blocking AMPK pathway (Fig. 4f, g), which indicated that NOD2 exerted the anti-tumor effect through effective activation of AMPK pathway. Moreover, activation of AMPK pathway and suppression of mTORC1 pathway induced by NOD2 were further verified in DEN/CCl_4_ induced HCC animal model and xenograft HCC model (Fig. 4h, i), which was in consistence with the in vitro study. Altogether, these data indicated that NOD2 exerted its anti-tumor effect on HCC cells through its effective activation of AMPK pathway.

### NOD2-induced autophagy-mediated apoptosis of HCC cells

In this study, we showed that NOD2 activated AMPK pathway and further suppressed subsequent mTORC1 pathway. Because inhibition of mTORC1 pathway is known to induce autophagy^18^, thus we are further interested in defining the effect of NOD2 on autophagy of HCC cells. The expression level of ATG proteins including Beclin-1, ATG12, ATG5, ATG7, ATG3, and ATG16L1, as well as the lipid form of LC3 were detected to reflect the activation status of autophagy in HCC cells. As expected, overexpression of NOD2 promoted the expression level of ATG proteins and lipid form of LC3 in HCC cells (Supplementary Figure 2 A), while knockdown of NOD2 inhibited the expression level of ATG proteins and lipid form of LC3 (Supplementary Figure 2B). These data showed that NOD2 significantly induced autophagy of HCC cells.

The consequence of autophagy activation was controversially reported^19^, thus we are further interested in defining whether autophagy induced by NOD2 finally leads to cell death of HCC cells. Annexin V-PI staining showed that overexpression of NOD2 could significantly induce apoptosis of HCC cells (Supplementary Fig. 2C). Further investigation demonstrated that NOD2-induced activation of Caspase 9-Caspase 3 mediated endogenous apoptosis pathway (Supplementary Figure 2D, E). To further verify whether the apoptosis of HCC cells was dependent on autophagy, we synthesized two specific SiRNAs against ATG5 (Si-ATG5) to inhibit the autophagy process. Our data demonstrated that when autophagy was blocked, the apoptosis of HCC cells was also significantly inhibited, which indicated that apoptosis of HCC cells induced by NOD2 was mediated by autophagy (Supplementary Fig. 2F). Besides, we also demonstrated that NOD2-induced autophagy-mediated apoptosis in DEN/CCl_4_ induced HCC model and xenograft HCC model (Supplementary Figure 2G, H). Altogether, these data indicated that NOD2 exerted the anti-tumor effect on HCC cells through its activation of autophagy-mediated apoptosis.

### NOD2 significantly enhanced the chemosensitivity of HCC cells to sorafenib, lenvatinib and 5-FU

Resistance to chemotherapy is the major obstacle to improve the prognosis of advanced HCC patient. NOD2 exerted its anti-tumor effect through activation of AMPK pathway, and the latter is recognized to be involved in regulating the response to chemotherapy^20–22^; thus we were interested in defining whether NOD2 has any effect on the response of HCC cells to chemotherapy. To elucidate this issue, we tested the effect of NOD2 on the response of HCC cells to three chemotherapeutic drugs: sorafenib, lenvatinib and 5-FU. Sorafenib, a tyrosine kinase inhibitor (TKI), was the FDA-approved first-line drug for advanced HCC patients^23^; while another TKI, lenvatinib has also been approved by FDA as a new first-line treatment for HCC in August 2018. 5-FU is a widely used anti-tumor drug for multiple cancers^24^, and it is also commonly used in the clinical treatment of HCC^25^. These three types of chemotherapeutic drugs are commonly used for the HCC patients and we were interested in defining the regulatory effect of NOD2 on the response of HCC cells to these chemotherapy drugs.

**Fig. 5:**
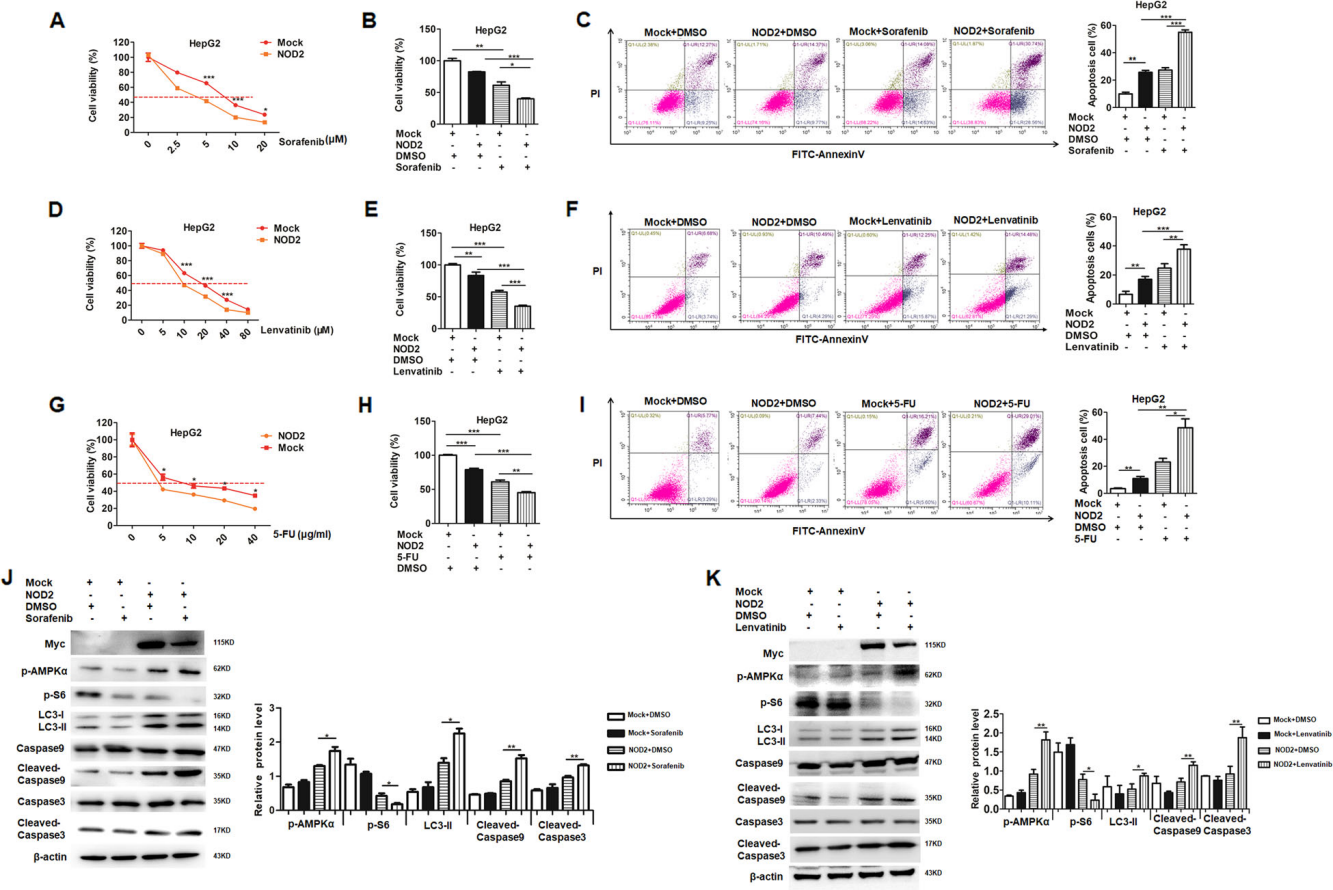
NOD2 significantly enhanced the chemosensitivity of HCC cells to sorafenib, lenvatinib and 5-FU. **a** HepG2 cells were plated in 96-well plate at the density of 1 × 10^4^ cells/well. After being cultured overnight, the cells were transfected with Myc-NOD2 plasmid or mock control plasmid. The transfected cells were further treated with different dosages (0 μM, 2.5 μM, 5 μM, 10 μM, and 20 μM) of sorafenib. 48 h after the treatment, CCK-8 assay was used to detect the cell viability. **b** HepG2 cells were plated in 96-well plate before being transfected with Myc-NOD2 or mock control. HCC cells were treated with sorafenib (5 μM), and the cells treated with the same volume of DMSO acted as a vehicle control. Cell viabilities were detected by CCK-8 assay after the cells were treated with sorafenib for 48 h. **c** HepG2 cells were plated in 6-well plate before transfected with NOD2 plasmid or mock control plasmid. HCC cells were treated with sorafenib (5 μM), and the cells treated with the same volume of DMSO acted as a control group. Annexin V/PI staining was used to detect cell apoptosis after the treatment with sorafenib for 48 h. **d** HepG2 cells were plated in 96-well plate at the density of 1 × 10^4^ cells/well. After being cultured overnight, the cells were transfected with Myc-NOD2 plasmid or mock control plasmid. The transfected cells were further treated with different dosages (0 μM, 5 μM, 10 μM, 20 μM, 40 μM, and 80 μM) of lenvatinib. 48 h after the treatment, CCK-8 assay was used to detect the cell viability. **e** HepG2 cells were plated in 96-well plate before being transfected with Myc-NOD2 plasmid or mock control plasmid. HCC cells were treated with lenvatinib (10 μM), and the cells treated with the same volume of DMSO acted as a vehicle control. Cell viabilities were detected by CCK-8 assay after the cells were treated with lenvatinib for 48 h. **f** HepG2 cells were plated in 6-well plate before being transfected with NOD2 plasmid or mock control plasmid. HCC cells were treated with lenvatinib (10 μM), and the cells treated with the same volume of DMSO acted as a control group. Annexin V/PI staining was used to detect cell apoptosis after the treatment with lenvatinib for 48 h. **g** HepG2 cells were plated in 96-well plate at the density of 1 × 10^4^ cells/well. After being cultured overnight, the cells were transfected with Myc-NOD2 plasmid or mock control plasmid. The cells were further treated with different dosages (0 μg/ml, 5 μg/ml, 10 μg/ml, 20 μg/ml and 40 μg/ml) of 5-FU. 48 h after the treatment, CCK-8 assay was performed to detect the cell viabilities. **h** HepG2 cells were plated in the 96-well plate before being transfected with NOD2 plasmid or mock control plasmid. HCC cells were treated with 5-FU (10 μg/ml) for 48 h before CCK-8 assay was performed to detect the cell viabilities. **i** HepG2 cells were plated in 6-well plate before being transfected with NOD2 plasmid or mock control plasmid. The transfected cells were treated with 5-FU (10 μg/ml) for 48 h before apoptosis was analyzed by Annexin V/PI staining. **j, k** HepG2 cells were transfected with Myc-NOD2 plasmid or mock control plasmid, followed by further treatment with sorafenib (5 μM) or lenvatinib (10 μM) for 48 h. Western blot assay was performed to detect the activation of AMPK pathway and mTORC1 pathway. Apoptosis and autophagy related markers of the sorafenib (**j**) or lenvatinib (**k**) treated HCC cells were also detected by western blot. *P < 0.05, **P < 0.01, ***P < 0.001 for statistical analysis of the indicated groups. Presented figures are representative data from three independent experiments.

Our data showed that exogenous overexpression of NOD2 could significantly increase the chemosensitivity of HCC cells to sorafenib (Fig. 5a), lenvatinib (Fig. 5d), and 5-FU treatment (Fig. 5g), and further investigation showed NOD2 significantly promoted the therapeutic effect of sorafenib (Fig. 5b), lenvatinib (Fig. 5e) and 5-FU (Fig. 5h) on HCC cells. Besides, NOD2 significantly increased the apoptosis rate of HCC cells after treatment with sorafenib (Fig. 5c), lenvatinib (Fig. 5f) and 5-FU (Fig. 5i).

To further define the molecular mechanism involved in the regulation of HCC cell by NOD2 to chemotherapy, we did western blot assay and our data showed that NOD2 significantly promoted the effect of sorafenib and lenvatinib by activating AMPK pathway and inhibiting mTORC1 pathway, followed by subsequent induction of autophagy-mediated apoptosis (Fig. 5j, k). All these above data demonstrated that NOD2 significantly enhanced the chemosensitivity of HCC cells by its regulation of AMPK pathway.

### NOD2 promoted activation of AMPK signaling pathway through directly binding with LKB1-AMPKα complex

**Fig. 6:**
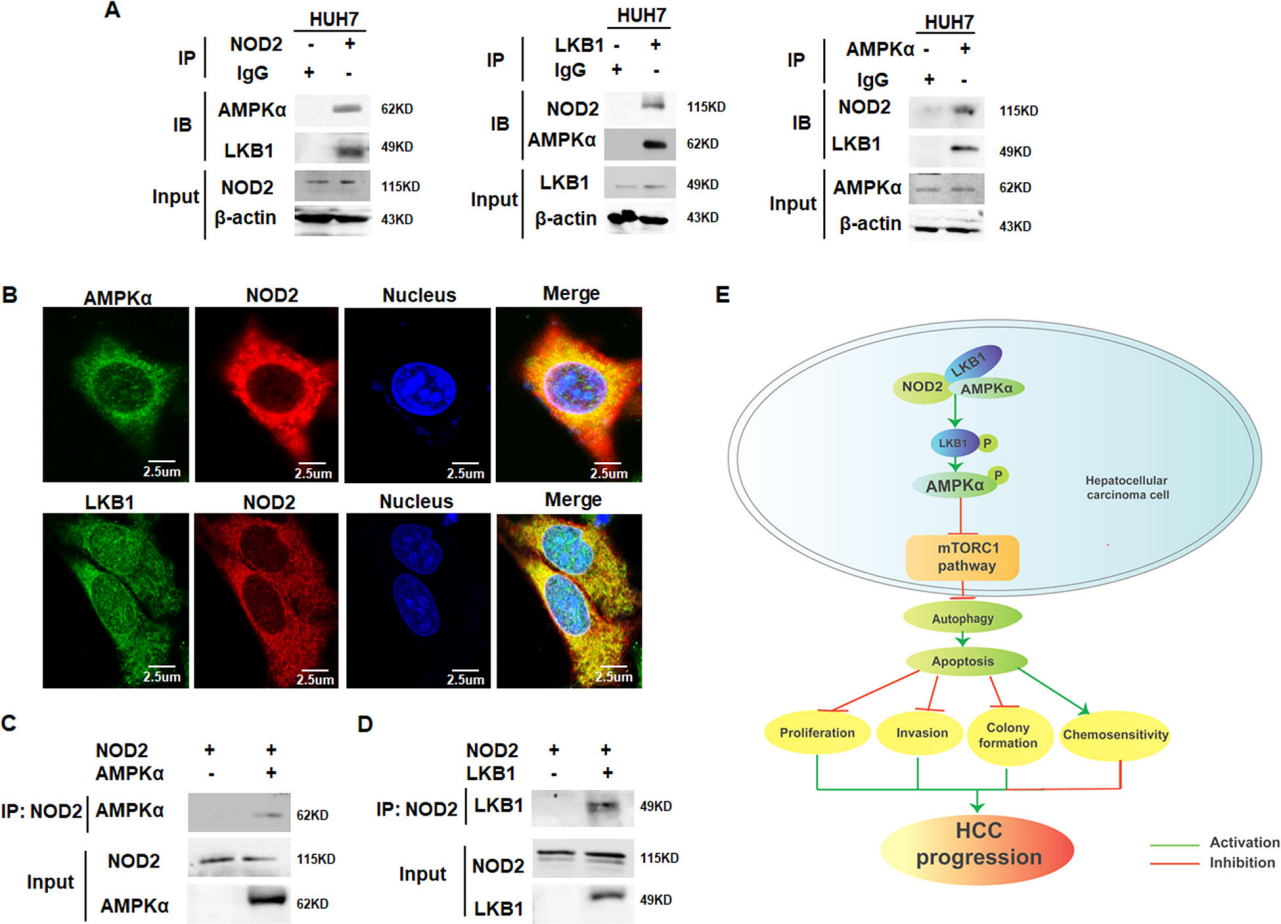
NOD2 promoted activation of AMPK signaling pathway through directly binding with LKB1-AMPKα complex. **a** HUH7 cells were cultured for 24 h before cell lysate was obtained for IP assay. The endogenous interaction between NOD2 and LKB1/AMPKα (left panel), LKB1 and NOD2/AMPKα (middle panel) as well as AMPKα and NOD2/LKB1 (right panel) were detected by IP assay by using the indicated antibodies. **b** HUH7 cells were cultured in 24-well plate for 24 h before the cells were fixed and stained with primary antibodies against NOD2 and AMPKα/LKB1, followed by staining with fluorescence-conjugated secondary antibodies (red for NOD2, green for AMPKα or LKB1). DAPI was used to stain the nuclei, and the co-localization between NOD2 and AMPKα/LKB1 was visualized as yellow fluorescence in the merged panel. **c**–**d** NOD2, AMPKα and LKB1 proteins were obtained from the in vitro translation system. NOD2 protein was mixed with AMPKα or LKB1 protein, and the direct binding between NOD2 and AMPKα (**c**), NOD2 and LKB1 (**d**) were detected by IP assay. All figures presented are representative data from at least three independent triplicate experiments. **e** The working model showing the role and mechanism of NOD2 in HCC cells. NOD2 directly binds with LKB-AMPKα complex and further activates LKB1-AMPKα pathway, which leads to inhibition of the mTORC1 pathway and activation of autophagy-mediated apoptosis in HCC cells; thus finally reverses the malignant behaviors of HCC cells and enhances the chemosensitivity of HCC cells to sorafenib, lenvatinib and 5-FU. Altogether, NOD2 acts as a tumor suppressor in HCC cells and loss of its expression in clinical HCC patients promotes HCC progression.

AMPKα is known to form a complex with its upstream activator LKB1 and be directly activated by LKB1^26^, thus we are interested in defining whether NOD2 could directly interact with AMPKα and LKB1. Co-immunoprecipitation (Co-IP) assay showed that NOD2 could bind with the endogenous LKB1 and AMPKα protein (Fig. 6a). In accordance with the current reports showing that AMPKα and LKB1 exerted their function by forming a complex, our data also showed the interaction between AMPKα and LKB1 protein (Fig. 6a). Immunofluorescence (IF) staining showed that NOD2 co-localized with both LKB1 and AMPKα in HCC cells (Fig. 6b), and the in vitro protein translation assay further verified that NOD2 directly interacted with both LKB1 and AMPKα (Fig. 6c, d). Altogether, these data indicated that NOD2 directly activated AMPK pathway by directly interacting with LKB1 and AMPKα, and thus forming a NOD2-LKB1-AMPKα complex in HCC cells.

Altogether, these data collectively verified that NOD2 exerted its anti-tumor effect by activating AMPK pathway and inducing autophagy-mediated apoptotic cell death of HCC cells, which finally reversed the malignant behaviors of HCC cells and enhanced the chemosensitivity of HCC cells to sorafenib, lenvatinib and 5-FU treatment (Fig. 6e).

## Discussion

NOD2 is well recognized as one of the pivotal innate immune sensors, which can sense pathogen infection and induce subsequent innate immune response^27–29^. Numerous recent studies indicated that innate immune sensors also critically participated in the progression of cancer, which indicated the potential potent link between innate immunity and malignancy^30–32^.

In this study, we investigated the role of NOD2 in the pathogenesis of HCC and showed that NOD2 deficiency promoted hepatocarcinogenesis, while overexpression of NOD2 inhibited tumorigenesis and reversed resistance to chemotherapy, which indicated an anti-tumor effect of NOD2 on HCC. We further validated NOD2 as a tumor suppressor as well as a potent regulator of chemosensitivity in HCC cells by directly regulating AMPK pathway.

AMPK is a highly-conserved master-regulator of numerous cellular processes, which is highly activated during stress. Because AMPK is located at the center of multiple established tumor suppressor networks including LKB1, TSC1–TSC2 complex and p53, it is recognized to play a pivotal role in cancer progression^3^. Deficiency of AMPK pathway is known to be correlated with cancer progression in several types of cancer^33–35^, thus effective activation of AMPK pathway is well recognized as an efficient cancer manipulation strategy. In this study, we showed for the first time that NOD2 was an effective direct activator of AMPK pathway in HCC cells, which indicated its great potential for the manipulation of cancer. HCC is recognized to be highly resistant to the chemotherapeutic drugs^36^, which results in its incontrollable development and lack of effective treatment for advanced disease. There is a desperate need to overcome chemoresistance and inhibit cancer progression for HCC patients. Since dysregulation of AMPK pathway is usually involved in resistance to chemotherapeutic drugs in several types of cancer^4–6^, thus it is plausible to expect that NOD2, a direct activator of AMPK pathway to have a regulatory role in resistance to chemotherapy. Therefore we test the effect of NOD2 on the response of HCC cells to chemotherapeutic drugs, and we showed that NOD2 could significantly increase the chemosensitivity of HCC cells to the most commonly used chemotherapeutic drugs, including sorafenib, lenvatinib and 5-FU. Further investigation verified that NOD2 regulated chemosensitivity of HCC cells through its activation of AMPK pathway and further induction of apoptosis of HCC cells. Thus this study indicated a great potential for clinical application of NOD2 for enhancing the treatment efficiency of chemotherapeutic drugs in HCC.

The regulation of AMPK pathway by NOD2 has been defined in this study, and we are further interested in clarifying the molecular mechanism in this regulatory process. AMPK activation is known to be efficiently induced by forming a complex containing LKB1, a known tumor suppressor which is mutated or silenced in various cancers^37,38^. Many of the best understood functions of LKB1 are attributable to its ability to activate AMPKα, which further exerts its effect on multiple cellular processes including cell growth^39^. Immunoprecipitation assay showed that NOD2 could bind with both AMPKα and LKB proteins; and further in vitro protein translation assay defined that NOD2 could directly interact with both AMPKα and LKB1 proteins in vitro. Thus our data demonstrated that NOD2-induced activation of AMPK pathway by directly interacting with both AMPKα and LKB1 and further forming a NOD2–LKB1–AMPKα complex to exert their anti-tumor effect on HCC cells. Different from the report showing that NOD2 indirectly activates AMPK by regulating immunity-related GTPase family M protein (IRGM) in innate immune response^40^, our study demonstrated for the first time that NOD2 directly interacted with AMPKα and further formed a NOD2-LKB1-AMPKα complex to induce activation of AMPK pathway.

To further clarify the consequence of NOD2-induced AMPK pathway activation, we detected its downstream mTORC1 pathway, and possible subsequent phenotypes including autophagy and apoptosis. Our data showed that NOD2 significantly inhibited mTORC1 pathway through its regulation of AMPK pathway, which finally led to autophagy-mediated apoptosis of HCC cells. Autophagy is a cellular self-degradation process in response to stress, and it has been extensively studied in recent years^41,42^. Though both pro-survival and pro-apoptosis role of autophagy have been reported in cancer^43^, it is recognized in recent years that the development of HCC is mainly attributed to deficiency of autophagy of the hepatic parenchymal cells^44^. The relationship between autophagy and apoptosis varies in different studies, and their complicated regulation is involved in the progression of cancer^43^. In this study, we showed that NOD2 could induce autophagy and further promote autophagy-mediated apoptosis of HCC cells. Thus we demonstrated a regulatory role of NOD2 in the process of autophagy and apoptosis of HCC cells, and therefore defined a pro-apoptotic death effect of NOD2 on HCC cells.

The role of NOD2 in cancer immunity is controversially reported^12,45,46^, and it varies depending on the types of cancer. NOD2 is an important component of the innate immune system and it constitutes an interesting regulatory target in terms of strengthening the immune response against cancer cells. Therefore several types of NOD2 agonists were synthesized which exhibited favorable anticancer activity^12^. However, another study showed that NOD2 antagonist inhibited both NF-κB and MAPK inflammatory signaling, and played an adjuvant role to paclitaxel (PTX) to suppress Lewis lung carcinoma (LLC) growth^45^. In addition, it has been reported that MDP analogs can inhibit the recruitment of myeloid-derived suppressor cells (MDSCs) to exert their anti-tumor effect^47^. These discrepant reports may be caused by the different types of cancer and divergent context of the TME. NOD2 is reported to activated NF-κB and MAPK pathways in macrophages and certain types of cancers^46,48^, however in HCC cells, NOD2 mainly activated AMPK pathway and exerted its anti-tumor effect through AMPK pathway. The favorable effect of NOD2 on suppressing tumor and increasing chemosensitivity of HCC is reported here for the first time, which may indicate novel therapeutic strategy against HCC based on the modulation of NOD2.

In conclusion, we investigated the role of NOD2 in an integrate investigation system including animal model, cellular model and clinical specimen, and demonstrated that NOD2 acted as a tumor suppressor in HCC cells. We verified that NOD2 inhibited HCC progression and enhanced chemosensitivity through directly forming a NOD2-LKB1-AMPKα complex and further activating AMPK pathway, which finally led to autophagy-mediated apoptosis of HCC cells. Thus we identified NOD2 as a novel tumor suppressor and chemotherapeutic regulator in HCC cells for the first time, which indicated a potential therapeutic strategy for HCC by positive regulation of NOD2.

## Materials and methods

### HCC animal models

NOD2-/- (*n* = 7) and WT (*n* = 8) male mice were injected with DEN (100 mg/kg, i.p.) at the age of 6 weeks, followed by 12 injections of CCl_4_ (0.5 ml/kg, i.p.) to induce HCC animal model according to the published procedure^13^. The xenograft tumor models in nude mice were constructed in 5-week-old male BALB/c nude mice as described before^14^. 15 mice were used for construction of the loss-of-function animal model and 11 mice were used for the gain-of-function model. During the investigation of pathological analysis of the HCC mice model, two professional pathologists were blinded to the group allocation. All of the protocols carried out to mice followed the guidelines of the Institutional Animal Care and Use Committee, and were approved by the Medical Ethics Committee of Shandong University.

### Cell lines and cell culture

The HCC cell lines including HepG2 cells (RRID:CVCL-0027) and HUH7 cells (RRID: CVCL-0336) were obtained from the Cell Bank of Chinese Academy of Science (Shanghai, China) and were maintained in DMEM medium complemented with 10% heat-inactivated FBS, 100 U/ml penicillin, and 100 μg/ml streptomycin. All of the experiments were performed with mycoplasma-free cells. The cell line authentication reports were provided by HKGENE genetechnology co. LTD (Beijing, China) within one year.

### Small interference RNA, plasmids, and transfection

HCC cells were transfected with plasmid or small interference RNA (SiRNA) according to the previously described procedure^14^. All the cell lines were cytogenetically tested and authenticated before the cells were used. SiRNA sequences against NOD2 and ATG5 were synthesized by Sigma-Aldrich (Merk, Darmstadt, Germany). The pCMV-Myc-NOD2 plasmid was provided by Dr.Sung Ouk Kim from University of Western Ontario. The HA-LKB1 plasmid and Flag-AMPKα plasmid were purchased from SINO (Sino biotechnology company, Shanghai, China) and Vigene Biology (Jinan, Shandong, China), respectively.

### Proliferation, invasion, colony formation and apoptosis assay

The viability, invasion, and colony formation assays of HCC cells were performed as previously described^14^. Cell apoptosis was detected by fluorescence activated cell sorting (FACS) analysis by using an Annexin V-FITC/PI staining kit (#40914, Biolegend, San Diego, USA) according to the manufacturer’s instructions.

### Western blot, immunochemistry, and immunoprecipitation assay

Western blot and immunochemistry were performed and evaluated as previously described^14,49^. Primary antibodies including specific antibodies against NOD2 (20980-1-AP), HA (51064-2-AP) and β-actin (60008-1-Ig) were purchased from Protein-tech (Philadelphia, PA, USA). Primary antibodies against p-AMPKα (#2535), p-LKB1 (#3482), AMPKα (#5831), p-AMPKβ (#4181), p-mTOR (#5536), p-S6K1 (#9234), p-S6 (#4858), p-4E-BP1 (#2855), ATG3 (#3415), ATG5 (#12994), ATG7 (#8558), ATG12 (#4180), Beclin-1 (#3495), ATG16L1 (#8089), LC3B (#12741), Caspase3 (#9662) and Caspase9 (#9508) were purchased from Cell Signaling Technology (Danvers, MA, USA). Immunoprecipitation assay was performed as previously described^14^.

### Clinical specimens

To evaluate the levels of NOD2 in HCC patients, we detected the expression of NOD2 in paired HCC tissues and corresponding distal non-cancerous liver tissues from 229 HCC patients in the Department of Hepatobiliary Surgery of the Provincial Hospital Affiliated to Shandong University. Among these cases, 165 pairs of matched tissue samples were used for immunohistochemistry staining, and 64 pairs of matched cancer and non-cancerous liver tissues were used for western blot and qRT-PCR assay. Written informed consents were obtained from all patients before participation. All protocols were in accordance with the Helsinki Declaration and were approved by Shandong University Research Ethics Committee.

### qRT-PCR assay

Total RNA was extracted from liver tissues by TRIzol reagent (Invitrogen, Waltham, MA, USA), and qRT-PCR was performed as described before^10^. The primer sequences of NOD2 were as follows: 5′-3′ TGGTTCAGCCTCTCACGATGA; 3′-5′ CAGGACACTCTCGAAGCCTT. Relative NOD2 mRNA expression was normalized to β-actin.

### Immunofluorescence assay and in vitro protein translation assay

The immunofluorescence assay was performed as described before^15^. Antibody against NOD2 was purchased from Abcam (#ab31488, Cambridge, MA, USA), and the other antibodies were the same as used for western blot. The in vitro protein translation assay was performed by TNT Quick Coupled Transcription and Translation System (Promega, Madison, WI, USA) as previously described^15^.

### Statistical analysis

Data were presented as mean ± SD and analyzed with Prism GraphPad 5.0 (GraphPad Software, La Jolla, CA, USA) software. Categorical variables were analyzed by Χ^2^-tests, and continuous variables were analyzed using ANOVA and T test. Spearman rank correlation assay was used for analysis of categorical variables, and Person correlation test was used for analysis of continuous variables. All tests were two-tailed, and *P* < 0.05 was considered statistically significant.

## Supplementary information

Supplementary figure legends

Supplementary figure 1

Supplementary figure 2

Supplementary table legends

Supplementary table 1

Supplementary table 2
