# Supplementary material for: NOD2 inhibits tumorigenesis and increases chemosensitivity of hepatocellular carcinoma by targeting AMPK pathway
Source: Cell Death Dis. 2020 Mar 6;11(3):174. doi: 10.1038/s41419-020-2368-5 (PMC7060316; doi:10.1038/s41419-020-2368-5)
Supplement: Supplementary file 1 — Supplementary figure legends [file 41419_2020_2368_MOESM1_ESM.docx]

**Supplementary figure legends**

**Supplementary Figure 1. MDP activated NOD2 inhibits the malignant behaviors of HCC cells. (A)** HUH7 cells were plated in 96-well plate at the density of 6x10^3^ cells/well. After being cultured overnight, the cells were treated with MDP (10μg/ml) or control. 48h after the treatment, CCK-8 assay was performed to detect the cell viability. (B-C) HUH7 cells were treated with the MDP (10μg/ml) or control, and the invasion (B) and colony formation (C) capabilities of these transfected cells were detected and compared. Cells were treated with MDP every 24 hours.

**Supplementary Figure 2. NOD2 induced autophagy-mediated apoptosis of HCC cells. (A)** HepG2 cells were transfected with NOD2 plasmid and the autophagy related markers were detected by western blot. **(B)** HUH7 cells were transfected with Si-NOD2, and the autophagy related markers including Beclin-1, ATG12, ATG7, ATG5, ATG3, ATG16L1 and LC3 were detected by western blot. **(C)** HepG2 cells were transfected with NOD2 plasmid and further cultured for 24h before apoptosis assay by Annexin V-PI staining. **(D)** HepG2 cells were transfected with NOD2 plasmid and further cultured for 24h. Activation of caspase 9 and caspase 3 were detected by western blot. **(E)** HUH7 cells were transfected with Si-NOD2 and further cultured for 24h before western blot assay of caspase 9 and caspase 3. **(F)** NOD2 transfected HepG2 cells were co-transfected with Si-ATG5 to block autophagy in NOD2 overexpressed cells. Autophagy and apoptosis levels of the transfected cells were detected by western blot. **(G)** NOD2-/- and WT mice were injected with DEN and CCl4 to induce hepatocellular carcinoma as described before. Proteins were extracted from the livers of NOD2-/- and WT mice for western blot assay to detect the activation of autophagy and apoptosis. **(H)** HCC cells were injected to nude mice to construct the xenograft tumor models as described before. When visible tumor appeared, the mice were divided into NOD2 transfected group and mock group as described before. Proteins were extracted from the tumors of NOD2 and mock transfected tumors for western blot assay to detect the activation of autophagy and apoptosis. The densities of all bands were analyzed by Image J and statistically analyzed by GraphPad software. *P<0.05, **P<0.01, ***P<0.001 for statistical analysis of the indicated groups.
