# Supplementary figures and images for: NOD2 inhibits tumorigenesis and increases chemosensitivity of hepatocellular carcinoma by targeting AMPK pathway
Source: Cell Death Dis. 2020 Mar 6;11(3):174. doi: 10.1038/s41419-020-2368-5 (PMC7060316; doi:10.1038/s41419-020-2368-5)

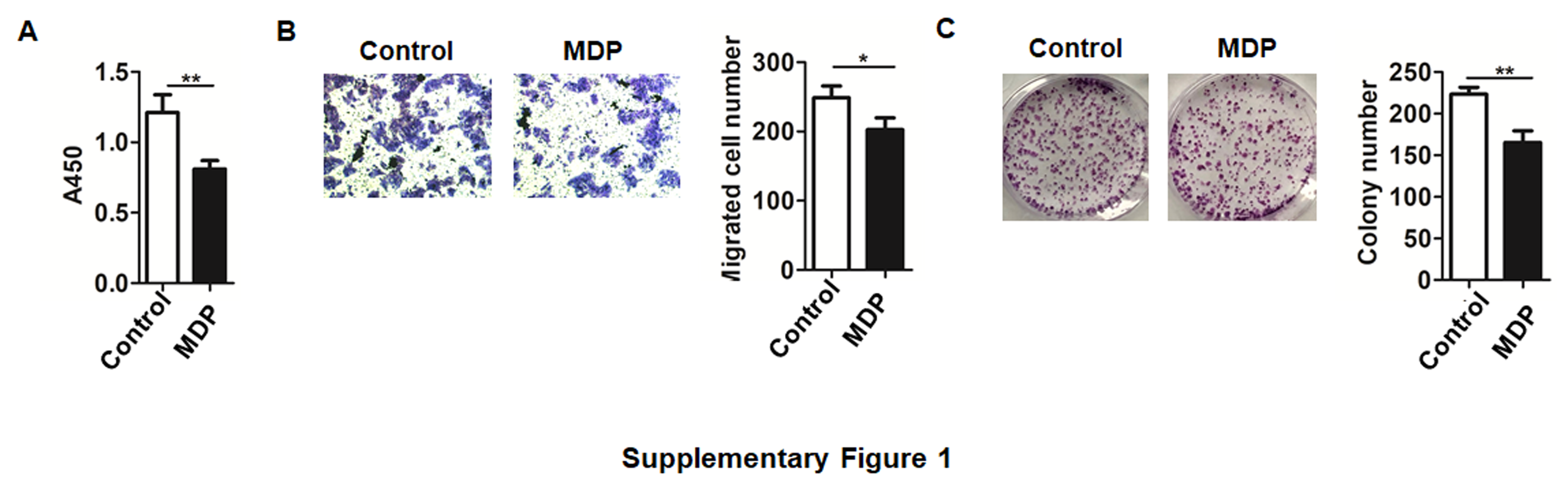

Supplement: Supplementary file 2 — Supplementary figure 1 [file 41419_2020_2368_MOESM2_ESM.tif]

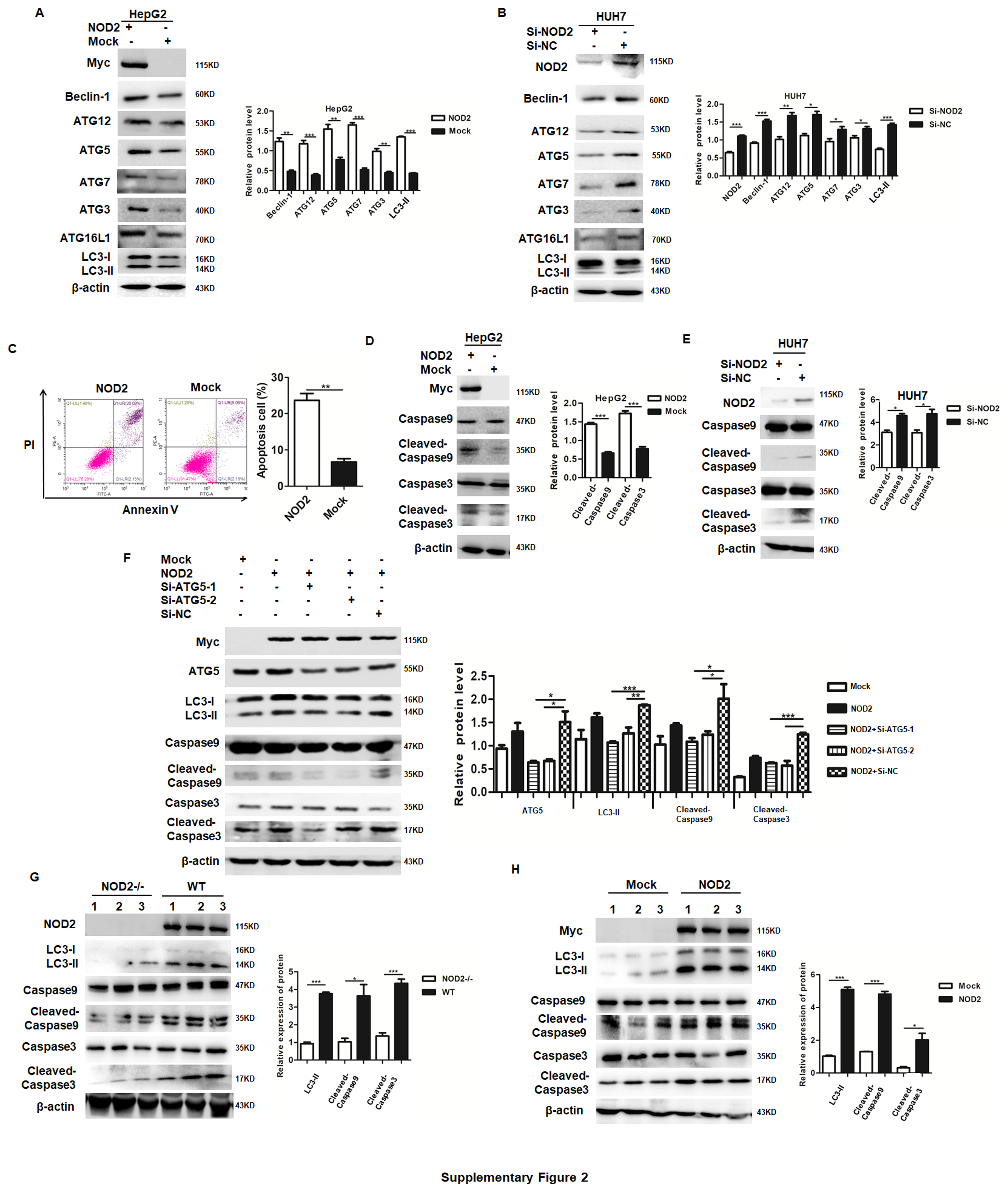

Supplement: Supplementary file 3 — Supplementary figure 2 [file 41419_2020_2368_MOESM3_ESM.tif]
