# Supplementary material for: NOD2 inhibits tumorigenesis and increases chemosensitivity of hepatocellular carcinoma by targeting AMPK pathway
Source: Cell Death Dis. 2020 Mar 6;11(3):174. doi: 10.1038/s41419-020-2368-5 (PMC7060316; doi:10.1038/s41419-020-2368-5)
Supplement: Supplementary file 4 — Supplementary table legends [file 41419_2020_2368_MOESM4_ESM.docx]

**Supplementary table 1**

**Clinicopathological characteristics of the investigated HCC patients**

**Supplementary table 2**

**Expression of NOD2 in liver cancer tissues compared with non-cancerous liver tissue**
