# Supplementary material for: NOD2 inhibits tumorigenesis and increases chemosensitivity of hepatocellular carcinoma by targeting AMPK pathway
Source: Cell Death Dis. 2020 Mar 6;11(3):174. doi: 10.1038/s41419-020-2368-5 (PMC7060316; doi:10.1038/s41419-020-2368-5)
Supplement: Supplementary file 5 — Supplementary table 1 [file 41419_2020_2368_MOESM5_ESM.docx]

**Supplementary table 1**

**Clinicopathological characteristics of the investigated HCC patients**

|  |  | | | **Cohort 1**  **(n=165)** | | |  | | | **Cohort 2**  **(n=64)** | | |  |  |
| --- | --- | --- | --- | --- | --- | --- | --- | --- | --- | --- | --- | --- | --- | --- |
| **Characteristics** |  | | | **No. of patients (%)** | | |  | | | **No. of patients (%)** | | |  |  |
| **Gender** |  | | |  | | |  | | |  | | |  |  |
| Male |  | | | 137 (83.0%) | | |  | | | 54(84.4%) | | |  |  |
| Female |  | | | 28 (17.0%) | | |  | | | 10(15.6%) | | |  |  |
| **Age** |  | | |  | | |  | | |  | | |  |  |
| <57 |  | | | 81 (49.1%) | | |  | | | 30(46.9%) | | |  |  |
| ≥57 |  | | | 84 (50.9%) | | |  | | | 34(53.1%) | | |  |  |
| **Liver cirrhosis history** | |  | | |  | | |  | | |  | | |  |
| Yes |  | | | 55 (33.3%) | | |  | | | 51(79.7%) | | |  |  |
| No |  | | | 110 (66.7%) | | |  | | | 13(20.3%) | | |  |  |
| **TNM stages** |  | | |  | | |  | | |  | | |  |  |
| I |  | | | 70 (42.4%) | | |  | | | 31(48.4%) | | |  |  |
| II |  | | | 9 (5.5%) | | |  | | | 3(4.7%) | | |  |  |
| III |  | | | 66 (40.0%) | | |  | | | 21(32.8%) | | |  |  |
| IV |  | | | 20 (12.1%) | | |  | | | 9(14.1%) | | |  |  |
| **Regional lymph nodes** | | |  | | |  | | |  | | |  | | |
| N0 |  | | | 114 (69.1%) | | |  | | | 25(39.1%) | | |  |  |
| N1 |  | | | 51 (30.9%) | | |  | | | 39(60.9%) | | |  |  |
| **BCLC stages** |  | | |  | | |  | | |  | | |  |  |
| 0 |  | | | 6 (3.6%) | | |  | | | 14(21.9%) | | |  |  |
| A |  | | | 71 (43.0%) | | |  | | | 19(29.7%) | | |  |  |
| B |  | | | 43 (26.1%) | | |  | | | 8 (12.5%) | | |  |  |
| C |  | | | 44 (26.7%) | | |  | | | 23(35.9%) | | |  |  |
| D |  | | | 1 (0.6%) | | |  | | | 0 | | |  |  |
| **Distant metastasis** | | |  | | |  | | |  | | |  | | |
| No |  | | | 110 (66.7%) | | |  | | | 20(31.2%) | | |  |  |
| Yes |  | | | 55 (33.3%) | | |  | | | 44(68.8%) | | |  |  |
