# Supplementary material for: NOD2 inhibits tumorigenesis and increases chemosensitivity of hepatocellular carcinoma by targeting AMPK pathway
Source: Cell Death Dis. 2020 Mar 6;11(3):174. doi: 10.1038/s41419-020-2368-5 (PMC7060316; doi:10.1038/s41419-020-2368-5)
Supplement: Supplementary file 6 — Supplementary table 2 [file 41419_2020_2368_MOESM6_ESM.docx]

**Supplementary table 2**

**Expression of NOD2 in liver cancer tissues compared with non-cancerous liver tissue**

| **Cancer (%) Non-cancer(%) χ2 P-value** |
| --- |

**NOD2**

Low 61.2 (101/165) 28.5 (47/165) 38.84 0.000***

High 38.8 (64/165) 71.5 (118/165)

Note: Low= ‘-, +’, High= ‘++, +++’, ***P<0.001 by statistical analysis using χ2-test.
